# A Comparative Evaluation of Push-Out Bond Strength of Six Different Root Canal Sealers: An In-Vitro Study

**DOI:** 10.7759/cureus.56481

**Published:** 2024-03-19

**Authors:** Divya Dudulwar, Suvarna Patil, Siddhesh Bandekar, Madhuri Patil, Divya Gupta, Ruchika Gupta

**Affiliations:** 1 Department of Conservative Dentistry and Endodontics, D Y Patil Dental School, Pune, IND; 2 Department of Conservative Dentistry and Endodontics, Vasantdada Patil Dental College and Hospital, Sangli, IND; 3 Department of Conservative Dentistry and Endodontics, Yogita Dental College and Hospital, Khed, Khed, IND; 4 Department of Conservative Dentistry and Endodontics, M. A. Rangoonwala College of Dental Sciences and Research Centre, Pune, Pune, IND

**Keywords:** smart seal, root canal sealer, propoints, procosol, gutta-percha

## Abstract

Background: Adhesiveness with radicular dentin is absent with gutta-percha, leading to microleakage and hence re-infection. Root canal sealer helps to achieve an adhesive interface between gutta-percha and root dentin thereby resisting the displacement forces during the functioning of teeth which is evaluated by the push-out test. The aim of this study is to compare the push-out bond strength and to assess the relative bond failure between dentin-sealer, sealer-main cone of (1) epoxy resin, (2) silicon, (3) mineral trioxide aggregate (MTA), (4) calcium hydroxide, (5) bioceramic, (6) zinc oxide eugenol containing root canal sealers.

Methodology: Sixty human permanent lower premolars with one root were collected, disinfected, and decoronated at cemento-enamel junction. Instrumentation was done with a K3 40,0.06 Ni-Ti rotary file and obturated using the main cone and sealer. Based on the sealer utilized, six groups were created: Group 1: AH-Plus, Group 2: RoekoSeal, Group 3: MTA Fillapex, Group 4: Apexit, Group 5: Smart Paste Bio, and Group 6: Procosol. One slice each was obtained from the coronal, middle, and apicalsections of all the obturated canals. Push-out bond strength and failure modes were studied. Statistics involved analysis of variance (ANOVA) followed by the post hoc Tukey test.

Results: All three sections exhibited the highest strength for Smart Paste Bio sealer and the least was for RoekoSeal. With all the sealers, the apical section had the highest strength followed by the middle and coronal.

Conclusion: The smart seal system was superior to all other sealers and displayed a good bond to dentin.

## Introduction

Three-dimensional debridement of the root canals eliminates the pathogenic microbes and establishes a total seal thereby preventing re-infection in periapical tissues and ensuring the success of the root canal treatment [1]. In spite of being the most widely used core filling material, gutta-percha lacks the spontaneous bonding to the dentin walls, hence to attain an ideal seal, it is always used along with a sealer. To prevent microleakage of bacteria, adhesion is essential between gutta percha, sealer material, and root dentin. This technique leaves behind two interfaces; core-sealer and dentin-sealer [2].

In an attempt to deal with the re-infection, root canal sealers should seal well and should have a good bond to the root dentin and gutta-percha so well that it resists the displacement forces occurring during the operative procedures or tooth functioning which may contribute to microleakage. The rationale of the study was to determine the bond strength and hence the adhesion of various root canal sealers to both gutta-percha and root dentin which is the clinical desirability in obtaining a hermetic seal that could prevent microbial microleakage. The thin-slice push-out test has been reported by Adams et al. in the literature to assess the resistance toward the displacement forces [3].

At different pH values and in the presence of different solubility mediums used, epoxy resin-based AH-Plus sealer has shown positive results and significantly lowest weight loss in water and artificial saliva [4,5]. RoekoSeal is reported to polymerize without shrinkage [6]. The most widely used calcium hydroxide has antibacterial and biological properties. Additionally, the mineral trioxide aggregate (MTA)-based sealer, MTA Fillapex used for root canal obturation too has shown remarkable outcomes [7].

One of the most latest developments in obturation materials is hydrophilic polymers. Absorption of residual moisture from the dentinal fluid and root canal space leads to lateral expansion of Smart Seal obturation points [8].

A push-out test was designed to gauge the bond of the sealers-main cone to the root canal wall. Using the stereomicroscope and scanning electron microscope, the failure modes were also analyzed.

The aim of this study was to compare the push-out bond strength and to assess the relative bond failure between dentin-sealer, sealer-main cone of (1) epoxy resin, (2) silicon, (3) MTA, (4) calcium hydroxide, (5) bioceramic, (6) zinc oxide eugenol containing root canal sealers.

The objectives were (a) to evaluate the push-out bond strength of (1) epoxy resin, (2) silicon, (3) MTA, (4) calcium hydroxide, (5) bioceramic, (6) zinc oxide eugenol containing root canal sealers; (b) to compare the push-out bond strength of (1) epoxy resin, (2) silicon, (3) MTA, (4) calcium hydroxide, (5) bioceramic, (6) zinc oxide eugenol containing root canal sealers; and (c) to assess the relative bond failure between the dentin-sealer and sealer-main cone with respect to all the sealers.

## Materials and methods

The study was approved by the Institutional Ethical Committee bearing number 820/2013-14. Lower second premolars (n=60) were included and teeth with open apex, root fracture, root caries, and root resorption were excluded in the study. The sample size was calculated by applying the formula {N= (Z a/2)^2^ * S^2^/d^2^, where N is the sample size, Z=1.96, S is Standard Deviation, and a=0.05} using OpenEpi Version 3.01 software (Open Source Epidemiologic Statistics for Public Health, www.OpenEpi.com). The teeth were cleaned with 5% NaOCl (sodium hypochlorite) followed by storage in distilled water.

**Figure 1:**
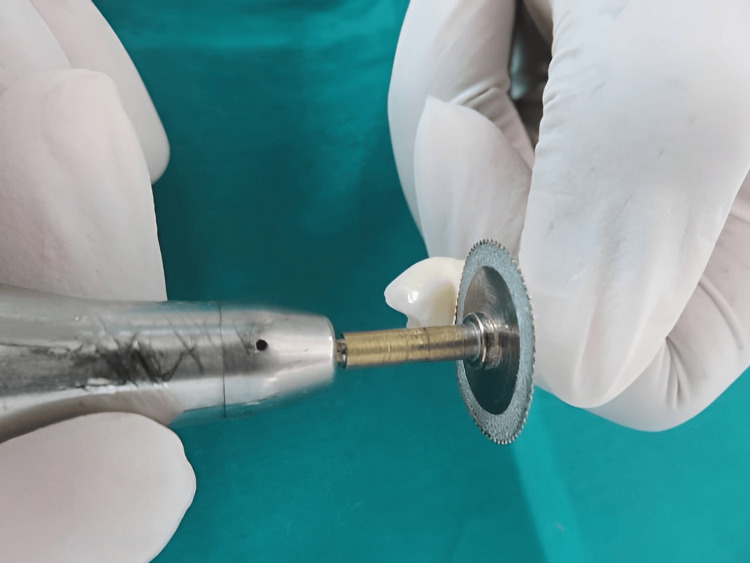
Decoronation of the sample at CEJ CEJ: cemento-enamel junction

The sectioning of teeth was done at the cemento-enamel junction (CEJ) using a disc in a low-speed contra-angled handpiece (Figure 1).

The patency of the canal was checked with a 10K file followed by biomechanical preparation using 0.06 taper K3 Ni-Ti rotary instruments to master apical rotary size 40 with 0.5mm working length less than the actual length. Irrigation was done using 5 ml of 5% NaOCl with a 26 gauge side bevel irrigating needle. Postpreparation, canals were rinsed with 5ml of 15% EDTA (ethylene diamine tetra acetic acid) followed by rinsing with 5ml distilled water. Random division of the teeth was done into six group. The composition of each sealer is discussed below (Table 1).

**Table 1:** Composition of sealers MTA: mineral trioxide aggregate

Materials	Composition	Manufacturer
AH-Plus	Paste A contains: Bisphenol-A epoxy resin, Bisphenol-F epoxy resin, Calcium tungstate, Zirconium oxide, Silica, Iron oxide pigments. Paste B contains: Dibenzyldiamine, Aminoadamantane, Tricyclodecane-diamine, Calcium tungstate, Zirconium oxide, Silica, Silicone oil.	Dentsply, Germany
RoekoSeal	Polydimethylsiloxane, Silicon oil, Paraffin-base oil, Platinum catalyst, Zirconium dioxide (radiopaque material).	Coltene Whaledent, Germany
MTA Fillapex	Salicylate resin, Diluting resin, Natural resin, Bismuth Trioxide, Nanoparticulated silica, MTA.	Angelus, Londrina, PR, Brazil
Apexit	Calcium salts (hydroxide, oxide, phosphate), Hydrogenized colophony, Disalicylate, Bismuth salts (oxide, carbonate), Highly dispersed silicon dioxide (silanized), Alkyl ester of phosphoric acid.	Ivoclar Vivadent
Smart Paste Bio - Pro Points	Resin, Bio Ceramic.	Smart Seal, UK
Procosol	Zinc oxide, Sodium borate, Barium sulphate, Bismuth subcarbonate, Hydrogenated Rosin, Eugenol.	Star Dental, Dentalez Group, USA

Group 1 (n=10): AH-Plus + Gutta-percha

Group 2 (n=10): RoekoSeal + Gutta-percha

Group 3 (n=10): MTA Fillapex + Gutta-percha

Group 4 (n=10): Apexit + Gutta-percha

Group 5 (n=10): Smart paste Bio + Smart points

Group 6 (n=10): Procosol + Gutta-percha

Experimental consistency in all the groups was maintained by following the passive fit single cone obturation technique.

Previous studies involving leakage tests on teeth obturated using a single cone method have shown differing results. The single cone method has been advocated for canals with parallel walls and the primary cone fits tightly in the apical one-third. With a matching taper single cone filling technique, this study showed similar mean push-out bond strength to previous studies that filled the root canal using a warm vertical condensation technique.

Cavit (3M ESPE, Maplewood, USA) was used for obtaining coronal seal and Lentulo Spiral (Dentsply Sirona, York, USA) for applying sealer. Storage of the samples was done at 37ºC and 100% humidity for 48 hours.

From each of the coronal, middle, and apical segments of each root sample, 1mm thick slices were obtained (30 slices/group). A stereomicroscopic examination of the samples was done to rule out the presence of voids. In case of void, the sample (sliced section) was discarded and a new slice was obtained. The stainless steel cylindrical plunger (0.5 mm wide) of the testing machine was placed over the main cone (Star Testing Systems, India. Model No. STS 248, Accuracy of the machine: ±1 Crosshead speed: 0.5 mm/minutes). The direction of force application was apical to coronal. The highest value recorded corresponded to the push-out bond strength.

Area under load= ½ × (Diameter of coronal aspect + Diameter of apical aspect) × thickness

Push-out strength (MPa) = Force (N) ÷ Area (mm)

Stereomicroscopic examination (“LYNX” Lawrence & Mayo) (20X) regarding the mode of failure amongst the samples revealed adhesive failure at the Dentin/Sealer (D/S) interface, combination adhesive failure at both the D/S and Sealer/Main cone (S/M) interface or mixed failure in both adhesive and cohesive modes. Specimens were further sectioned vertically, mounted on copper studs, coated with platinum, and were evaluated under Field Emission Gun Scanning Electron Microscopy (FEG-SEM) (400X).

Statistical analysis

One-way analysis of variance (ANOVA) test followed by Tukey post hoc test with significance p<0.05 analyzed the data.

Intergroup comparison

(1) Coronal Section

The push-out bond strength was highest for Group 5 (Smart Paste Bio) compared to all other groups which are followed by Group 1 (AH-Plus), Group 4 (Apexit), Group 6 (Procosol), Group 3 (MTA Fillapex), Group 2 (RoekoSeal) with the mean values of push-out bond strength of 4.02 MPa for Group 5 (Smart Paste Bio) and 0.05 MPa for Group 2 (RoekoSeal). Highly significant differences in push-out bond strength were noticed between groups and within groups (p<0.05). Intergroup comparison with respect to push-out bond strength when done by Tukey’s multiple post hoc procedures has shown highly significant difference (p<0.05) except Group 2 (RoekoSeal) when compared with Group 3 (MTA Fillapex) and Group 4 (Apexit) when compared with Group 6 (Procosol) was not significant.

(2) Middle Section

The push-out bond strength was highest for Group 5 (Smart Paste Bio) compared to all other groups which are followed by Group 1 (AH-Plus), Group 4 (Apexit), Group 6 (Procosol), Group 3 (MTA Fillapex), Group 2 (RoekoSeal) with the mean values of push-out bond strength of 5.60 MPa for Group 5 (Smart Paste Bio) and 0.16 MPa for Group 2 (RoekoSeal). Highly significant differences were noticed between groups and within groups (p<0.05). Intergroup comparison with respect to push-out bond strength when done by Tukey’s multiple post hoc procedures has shown highly significant difference (p<0.05) except when Group 2 (RoekoSeal) was compared with Group 3 (MTA Fillapex), Group 3 (MTA Fillapex) was compared with Group 6 (Procosol) and Group 4 (Apexit) was compared with Group 6 (Procosol) was not significant.

(3) Apical Section

The push-out bond strength was highest for Group 5 (Smart Paste Bio) compared to all other groups which are followed by Group 1 (AH-plus), Group 4 (Apexit), Group 6 (Procosol), Group 3 (MTA Fillapex), Group 2 (RoekoSeal) with the mean values of push-out bond strength of 7.89 MPa for Group 5 (Smart Paste Bio) and 0.27 MPa for Group 2 (RoekoSeal). Highly significant differences were noticed between groups and within groups (p<0.05). Intergroup comparison with respect to push-out bond strength when done by Tukey’s multiple post hoc procedures has shown a highly significant difference (p<0.05) for all groups.

Intragroup comparison

(1) Group 1 (AH-Plus)

The push-out bond strength was highest for the apical section with a mean value of 4.51 MPa as compared to the middle section and coronal section having mean values of 4.36 MPa and 2.20 MPa respectively. Pair-wise comparison for all three sections with respect to push-out bond strength when done by Tukey’s multiple post hoc procedures has shown a highly significant difference (p<0.05) except for the middle section when compared with the apical section was not significant.

(2) Group 2 (RoekoSeal)

The push-out bond strength was highest for the apical section with a mean value of 0.27 MPa as compared to the middle section and coronal section having mean values of 0.16 MPa and 0.05 MPa respectively. Pair-wise comparison for all three sections with respect to Push-out bond strength when done by Tukey’s multiple post hoc procedures has shown a highly significant difference only when the coronal section was compared with the apical section (p<0.05), while it was not significant when coronal section compared with the middle section and middle section when compared with apical section.

*(3) Group 3 (MTA Fillapex* *)*

The push-out bond strength was highest for the apical section with a mean value of 0.85 MPa as compared to the middle section and coronal section having mean values of 0.71 MPa and 0.35 MPa respectively. Pair-wise comparison for all three sections when done by Tukey’s multiple post hoc procedures has shown a highly significant difference (p<0.05) except when the middle section was compared with the apical section it was not significant.

(4) Group 4 (Apexit)

The push-out bond strength was highest for the apical section with a mean value of 1.89 MPa as compared to the middle section and coronal section having mean values of 1.76 MPa and 1.14 MPa respectively. Pair-wise comparison for all three sections with respect to push-out bond strength when done by Tukey’s multiple post hoc procedures has shown a highly significant difference (p<0.05) except when the middle section was compared with the apical section it was not significant.

(5) Group 5 (Smart Paste Bio)

The push-out bond strength was highest for the apical section with a mean value of 7.89 MPa as compared to the middle section and coronal section having mean values of 5.60 MPa and 4.02 MPa respectively. Pair-wise comparison for all three sections with respect to push-out bond strength when done by Tukey’s multiple post hoc procedures has shown a highly significant difference (p<0.05) for all three sections.

(6) Group 6 (Procosol)

The push-out bond strength was highest for the apical section with a mean value of 1.35 MPa as compared to the middle section and coronal section having mean values of 1.29 MPa and 0.93 MPa respectively. Pair-wise comparison for all three sections with respect to push-out bond strength when done by Tukey’s multiple post hoc procedures has shown a highly significant difference only when the coronal section was compared with the apical section (p<0.05) while it was not significant when the coronal section was compared with the middle section and middle section when compared with apical section.

## Results

**Figure 2:**
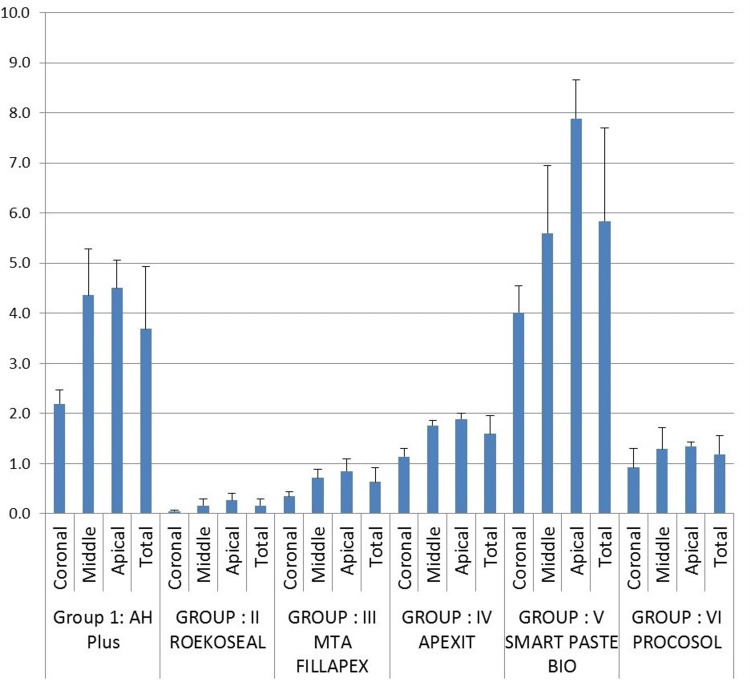
Mean push-out bond strength compared between all six groups in all three segments

The apical section had the highest push-out bond strength followed by the middle and coronal (Figure 2).

For AH-Plus sealer, the difference was significant between apical and coronal, middle and coronal sections with p<0.05. No significant difference was observed between the middle and apical sections.

A similar finding was noted with MTA Fillapex sealer and Apexit sealer. In the case of Procosol and Roekoseal sealer, the difference (p<0.05) was observed only between coronal and apical sections. Smart Paste Bio sealer presented a significant difference (p<0.05) amongst all the sections. In the case of the apical section, all the sealers presented a significant difference (p>0.05) (Table 2).

**Table 2:** Intergroup and intragroup comparison between all sections with respect to push-out bond strength *Significance at p<0.05

Intragroup Comparison				Intergroup Comparison				
Groups	Section		Significance (p)	Groups		Coronal section significance (p)	Middle section significance (p)	Apical section significance (p)
Group 1	Coronal	Middle	0.0001*	Group 1	Group 2	0.0001*	0.0001*	0.0001*
		Apical	0.0001*		Group 3	0.0001*	0.0001*	0.0001*
	Middle	Apical	0.8623		Group 4	0.0001*	0.0001*	0.0001*
Group 2	Coronal	Middle	0.0787		Group 5	0.0001*	0.0030*	0.0030*
		Apical	0.001*		Group 6	0.0001*	0.0001*	0.0001*
	Middle	Apical	0.1206	Group 2	Group 3	0.256	0.496	0.027*
Group 3	Coronal	Middle	0.001*		Group 4	0.0001*	0.0001*	0.001*
		Apical	0.001*		Group 5	0.0001*	0.0001*	0.001*
	Middle	Apical	0.2372		Group 6	0.0001*	0.008*	0.001*
Group 4	Coronal	Middle	0.001*	Group 3	Group 4	0.0001*	0.017*	0.0001*
		Apical	0.001*		Group 5	0.0001*	0.0001*	0.0001*
	Middle	Apical	0.080		Group 6	0.0001*	0.446	0.0001*
Group 5	Coronal	Middle	0.002*	Group 4	Group 5	0.0001*	0.0001*	0.0030*
		Apical	0.001*		Group 6	0.6035	0.0648	0.0001*
	Middle	Apical	0.001*	Group 5	Group 6	0.0001*	0.0001* 0.0001*	
Group 6	Coronal	Middle	0.057					
		Apical	0.023*					
	Middle	Apical	0.912					

Push-out bond strength for the coronal, middle, and apical sections was in the following descending order:

Smart Paste Bio > AH-Plus > Apexit > Procosol> MTA Fillapex > RoekoSeal

**Figure 3:**
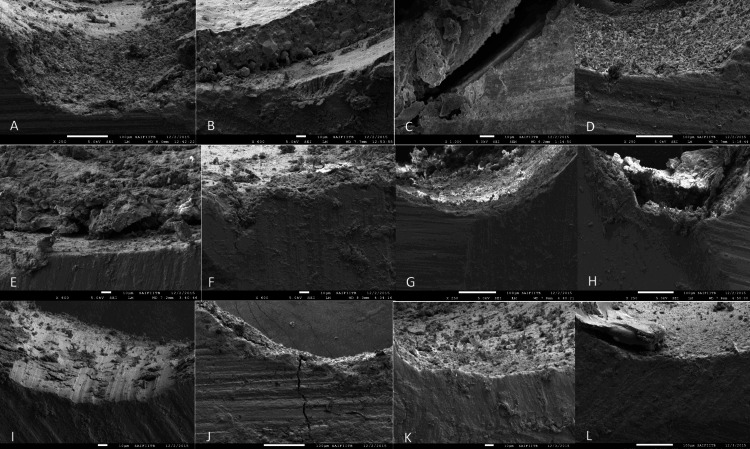
SEM images displaying failure modes AH-Plus - (A) Mixed failure in both adhesive and cohesive modes; (B) Combination failure at both D/S and S/M interface; Roekoseal - (C) Combination adhesive failure at both the D/S and S/M interface; (D) Adhesive failure at D/S interface; MTA Fillapex - (E) Combination failure at both the D/S and S/M interface; (F) Adhesive failure at D/S interface; Apexit - (G) Mixed failure in both cohesive and adhesive modes; (H) Combination failure at both the D/S and S/M interface; Smart Seal - (I) and (J) Mixed failure in both cohesive and adhesive modes; Procosol - (K) adhesive failure at D/S interface; (L) Mixed failure in both cohesive and adhesive modes D/S: Dentin/Sealer, S/M: Sealer/Main, SEM: Scanning Electron Microscopy

At 20X magnification, the adhesive failure at the D/S interface was highest with AH-Plus sealer (83%), combination adhesive failure at both the D/S and S/M interface was highest with MTA Fillapex sealer (63%) and mixed failure in both adhesive and cohesive modes was highest with Smart Paste Bio sealer (100%). The failure modes at 20X magnification under Stereomicroscope and 400X magnification under FEG-SEM are presented in Table 3 and Figure 3.

**Table 3:** Failure modes under 20X and 400-4000X magnification in all groups D/S: Dentin/Sealer

Groups	Under 20X magnification			Under 400-4000X magnification
	Adhesive failure at D/S interface	Combination adhesive failure at both the D/S and S/M interface	Mixed failure in both adhesive and cohesive modes	
Group 1	0%	17%	83%	- In combination failure: Complete debonding between sealer layer-dentin; gutta-percha sealer. - In mixed failure: Sealer tags covering dentinal tubules; main cone debonded completely.
Group 2	70%	30%	0%	- In D/S interface failure: Sealer tags still covering dentin (no complete debonding from dentin). - In combination failure: Complete debonding between sealer layer-dentin, gutta-percha-sealer.
Group 3	37%	63%	0%	- In combination failure: Debonding between sealer layer-dentin, gutta-percha-sealer (complete debonding). - In D/S interface failure: Sealer tags still covering dentin (no complete debonding from dentin).
Group 4	0%	20%	80%	- In combination failure: Debonding between sealer layer-dentin, gutta-percha-sealer (complete debonding). - In mixed failure: Complete debonding between sealer layer-dentin, gutta-percha-sealer.
Group 5	0%	0%	100%	- In mixed failure: Debonded sealer from the dentin surface with some sealer tags left on the dentin surface; pro-points debonded from the sealer.
Group 6	47%	0%	53%	- In D/S interface failure: Sealer tags still covering dentin (no complete debonding from dentin). - In mixed failure: Debonding of sealer completely at some places; bonded sealer to gutta-percha and dentin.

## Discussion

The resistance of the sealer material towards displacement due to the functional forces is essential to prevent microleakage and re-infection post-root canal therapy. The current study supplemented 5% NaOCl and 15% EDTA for irrigation to obtain the removal of inorganic debris and facilitate improved sealer penetration into the dentinal tubules thereby enhancing sealer-dentin contact.

This study demonstrated the highest push-out bond strength at apical sections compared to the middle and coronal third sections, as stated by Baldissera R et al. [9] the slant height is inversely proportional to bond strength. Thus, with geometrical characteristics unfavorable to dislodgement, the coronal and middle thirds slices exhibited lower mean strengths than apical thirds, which have geometrical characteristics favorable to dislodgment. Variation in the push-out bond strength might be attributed to the circular root canal section of the apical portion while oval, trapezoidal, or even flattened sections of coronal and middle slices.

The FEG-SEM (400X) displayed AH-Plus remarkably bonded to the dentin. Its epoxide ring opens up, reacting with any exposed amino groups in collagen forming covalent bonds with them. Creep potential, long setting time, greater penetration into the micro-irregularities, and improved mechanical interlocking between the sealer and root dentin further enhance the resistance to displacement from dentin [5]. Studies have reported minimal shrinkage while setting and prolonged dimensional stability [10]. A resemblance in the results was observed with the studies reported by Ersahan S et al. [10] and Abada HM et al. [11].

RoekoSeal sealer demonstrated the least push-out bond strength. Combination adhesive failure at both the D/S and S/M interface presented complete debonding of gutta-percha and sealer under FEG-SEM, contributing to their low adhesion to both. The value of 0.66 MPa obtained in the present analysis was close to that reported by Abada HM et al. [11]. The lowest bond strength of RoekoSeal can be due to the negligible reaction between dentin and sealer and the polydimethylsiloxane which is the principal component [12] and the same was also reported by Ali N et al. [13]. While no chemical bond to dentin is formed, it stretches by 0.2% to support lateral canal filling [14,3].

At the surface facing the gutta-percha, the ragged sealer boundary emerged, showing the sealer's adhesion to dentin and gutta-percha. In the sealer itself, the presence of sealer projection over the surface of dentinal tubules signifies its coherent breakdown. These results are found in accordance with the Sagsen et al. study [15].

The cumulative adhesive failure at the D/S and S/M interface indicated a mixed failure in both; adhesive and cohesive modes for Apexit. Wenneberg and Orstavik mentioned in their study that calcium hydroxide-based root canal sealers show low bond strength because the reaction between calcium hydroxide and glycol salicylate forms an amorphous calcium disalicylate, which does not bond to dentin [16]. The results of the present study are consistent with Gaddala N et al. [17].

For Smart Paste Bio failure modes were not significant due to the cohesiveness of the sealer and adhesion between Pro Points and Smart Paste Bio. Under FEG-SEM, some surface portions of the debonded sealer and other parts of the sealer layers still adhering to dentin were noted. A sealer sheet with no tubules left exposed, showing strong adhesion between the sealer and dentin, which was fully filled with dentin tubules. The Smart-Seal device is hydrophilic in design and has a staggered setting time (4-10 hours), causing the Pro Point to become hydrated and swollen thus filling up the voids [18]. They contain a radiopaque hydrophilic polymer that expands up to 17% laterally upon absorbing water from the tooth and adapts to the shape of the canal [19].

According to Mathew ST et al.'s procosol which is a zinc-oxide Eugenol-based sealer exhibited weak dentin adhesion, but stronger adhesion to gutta-percha over calcium hydroxide and resin-based root canal sealers [20]. These findings were consistent with the outcomes of the current research. The low push-out bond strength of this sealer is due to the chelating activity during the setting. It softens the gutta-percha thereby creating an intertwined meshwork and increasing adhesion between the materials [21].

Our study depicted that not all the sealers penetrated inside the exposed dentinal tubules and the bond strength achieved was not higher for all the sealers that were able to penetrate inside the tubules. Current theories of dentin bonding mechanisms involve either chemical modification of the smear layer and bonding directly to it, or removal of the smear layer and bonding to subjacent tooth structures. Also, the sealer penetration into the dentinal tubules is related not only to the smear layer removal but also to the physical and chemical properties of the sealer itself. This agrees with the suggestion provided by Saleh et al. that penetration of sealer tags inside the tubules thus creating micromechanical retention is not the only important factor influencing the adhesion of sealers [22]. Rather tubular penetration is clearly dependent on the chemical and physical properties of the sealer.

Limitations and future scope of the study

In our study mandibular premolar teeth were used which usually have an oval canal, so the film thickness might not be completely uniform throughout the canal which eventually might have resulted in different failure modes at all three sections of the root. This variability reflects the clinical situation, where large differences may be encountered between teeth. These in-vitro models cannot faithfully reproduce the clinical conditions because the scenario is well controlled, the root dentin is not uniform and may vary from one tooth to another. The bond strength was checked within two weeks after the obturation was completed, but these results cannot be completely implemented clinically because the bond strength may vary after a few months or years due to which the failure may occur. Further studies are required to test this hypothesis based on the fact that the bond strength may vary after a few months or years due to which the failure may occur. The time duration might be altered.

## Conclusions

To avoid re-infection and failure, bonding of sealer to gutta-percha and root dentin is most important. Push-out bond strength was highest at apical followed by middle and coronal sections in all groups. Smart Paste Bio has the highest push-out bond strength in the apical section and the RoekoSeal has the lowest push-out bond strength for apical, middle, and coronal sections. The usage of hydrophilic sealer like Smart Paste Bio widens the range of achieving three-dimensional seal. Future research is expected to improve the physical properties of the sealer for minimizing microleakage in endodontic therapy.
